# Body Weight Prediction in Karayaka Lambs Using Morphometric Measurements: A Comparison of Regression and Machine Learning Approaches

**DOI:** 10.3390/ani16152288

**Published:** 2026-07-23

**Authors:** Lütfi Bayyurt

**Affiliations:** Department of Animal Science, Faculty of Agriculture, Tokat Gaziosmanpaşa University, Tokat 60240, Türkiye; lutfi.bayyurt@gop.edu.tr

**Keywords:** machine learning, gradient boosting, SHAP, principal component analysis, Karayaka, lamb

## Abstract

Body weight is one of the important phenotypic traits in sheep breeding for evaluating growth performance, planning flock management practices, and determining economic efficiency. In this study, biometric and machine learning approaches were jointly employed to predict body weight in Karayaka lambs using morphometric characteristics. The research material consisted of a total of 150 Karayaka lambs, including 75 males and 75 females, raised in a private enterprise located in the Erbaa district of Tokat province. The study evaluated body weight (BW), heart girth (HG), abdominal girth (AG), diagonal body length (DBL), body length (BL), withers height (WH), rump height (RH), hip width (HW), chest width (CW), and body condition score (BCS). Relationships among variables were examined using Pearson correlation analysis and principal component analysis (PCA). Multiple linear regression, Ridge Regression, LASSO regression, Random Forest, and Gradient Boosting algorithms were applied to predict body weight. Additionally, variable importance analysis and the SHAP (SHapley Additive Explanations) approach were utilized to enhance model interpretability. The results demonstrated significant differences between sexes in body weight and the majority of morphometric traits (*p* < 0.05). Correlation analysis revealed that abdominal girth and heart girth had the strongest associations with body weight. According to PCA results, the first principal component explained the majority of the total variance and represented overall body size. Among the evaluated machine learning models, the Gradient Boosting algorithm achieved the highest prediction performance, with a training R^2^ of 0.962 and a test R^2^ of 0.862, together with the lowest prediction errors (RMSE = 1.320 kg and MAE = 1.034 kg). The Random Forest model ranked second, achieving a test R^2^ of 0.828. Variable importance and SHAP analyses indicated that heart girth and abdominal girth were the most influential features in predicting body weight. In conclusion, morphometric traits can be effectively utilized to predict body weight in Karayaka lambs, and the Gradient Boosting algorithm represents a robust approach offering high accuracy and interpretability.

## Simple Summary

Accurate body weight measurement is essential for sheep management because it helps farmers make decisions about feeding, health care, breeding, and marketing. However, weighing scales are not always available, especially under field conditions. This study investigated whether simple body measurements could be used to estimate the body weight of Karayaka lambs. Several statistical and machine learning methods were compared, and the Gradient Boosting model provided the most accurate predictions. In addition, an explainable artificial intelligence technique was used to identify which body measurements contributed most to the prediction results. Heart girth and abdominal girth were found to be the most informative traits. These findings suggest that body weight can be estimated quickly and accurately using a few easily obtained measurements, reducing the need for specialized equipment. Although the models should be validated in larger and more diverse sheep populations before wider application, the proposed approach provides a practical and cost-effective tool that may support sheep farmers, breeders, and researchers in routine flock management and decision-making.

## 1. Introduction

Body weight is one of the major phenotypic characters in sheep breeding applicable to assess growth performance, design flock management practices and establish economic efficiency [1,2]. In addition, body weight is directly related to many breeding applications, such as monitoring feeding plans, calculating drug dosages, purchase timing, and carcass performance [3,4]. On the other hand, adopting direct measurement of body weight requires weighing devices that are often unavailable, which is a problem amidst small-scale business entities and large extensive breeding systems [1]. Therefore, the prediction of body weight based on easily measurable morphometric traits has been a topic of constant interest in livestock research for many years [5].

Biometric approaches are the most common methods for evaluating animal growth and development performance, which relate morphometric characteristics to body weight. It is quite simple to demonstrate that heart girth, abdominal girth, withers height, rump height and body length are significantly correlated with body weight [2,6,7] in several sheep breeds. Specifically, heart girth is described as one of the most important predictors of live body weight in many sheep populations from West African Dwarf [3], Hissardale [2], Corriedale and others [8]. Hence, prediction models based on body measurements can be used to estimate body weight in localities that do not have adequate weighing systems, since such estimates are rapid and cheaper [4].

Most studies concerning the estimation of live weight from morphometric characteristics have employed linear and multiple linear regression models [2,3,4,8]. However, many of these morphometric traits exhibit a high degree of correlation; this may lead to issues of covariation and, consequently, a complex relational structure among variables that classical regression methods alone may not fully elucidate. As a result, in recent years, multivariate approaches such as principal component analysis have become increasingly prevalent to uncover the underlying covariation structure among body measurements [9,10]. Both of these approaches contribute to understanding the structural interdependencies among variables and to developing improved models for more robust predictions.

Recent advances in machine learning have created new opportunities for improving body weight prediction studies in livestock species. Specifically, ensemble learning algorithms like Random Forest, Gradient Boosting and XGBoost have been popular among researchers in livestock over the last several years as a result of their ability to model nonlinear relationships and complex variable interactions [11,12]. Recent studies on different sheep breeds have shown that machine learning algorithms can lead to higher prediction accuracy in the prediction of body weight than conventional regression modeling approaches [13,14]. This evidence underscores the great promise of machine learning approaches for modeling complex biological properties such as body weight.

A major challenge in the deployment of machine learning models comes from the fact that while they are able to predict accurately most of the time, the underlying model is often not interpretable. Over the last few years, approaches to explainable artificial intelligence have emerged to make it possible to interpret complex models [15]. Based on this, the SHapley Additive exPlanations (SHAP) method has been gaining popularity in biological/agrarian data analysis, as it provides a quantitative measure of the contribution of each variable towards the model prediction [16]. Therefore, it is now feasible to show not only how well a model predicts, but also what the biological relevance of its decisions is.

Karayaka is one of the important indigenous sheep genotypes of Turkey, and it has been mainly raised in the Central and Eastern Black Sea Regions. The breed’s adaptability to environmental conditions and its resilience and regional importance for livestock farming have led to many studies being published on the growth and development characteristics [17,18]. While the associations between body weight, body measurements and carcass traits in Karayaka sheep have been investigated previously, large-scale studies that analyze the multivariate structure among morphometric traits and compare classical regression methods with state-of-the-art machine learning algorithms based on explainable artificial intelligence approaches concurrently are scarce.

Although numerous studies have investigated body weight prediction in sheep using conventional regression models or machine learning algorithms, direct comparisons between traditional statistical approaches and explainable machine learning techniques remain limited. Moreover, few studies have integrated SHAP-based explainable artificial intelligence to interpret the contribution of morphometric traits in body weight prediction, particularly in indigenous sheep breeds such as Karayaka. Therefore, the present study was designed not only to compare conventional regression methods with modern machine learning algorithms but also to improve model interpretability through explainable artificial intelligence.

This study aimed to predict body weight in Karayaka lambs using morphometric traits. The associations among morphometric traits were analyzed based on correlation and principal component analysis; also, the degree of multicollinearity was investigated, and multiple linear regression, Ridge Regression, LASSO regression models, Random Forests and Gradient Boosting models were compared in terms of body weight prediction. In addition, variable importance and SHAP were performed on the best-fitted model to explain how morphometric traits help body weight value prediction.

## 2. Materials and Methods

### 2.1. Animal Material and Body Measurements

The materials used in this study were collected at a private sheep breeding company located in the Erbaa district of Tokat province, which is one of the provinces located in Turkey’s Black Sea Region. The dataset consisted of body weight and morphometric measurements collected during the 2025 breeding season. The subjects of the study were 150 Karayaka lambs aged between 160 and 200 days (75 males and 75 females). Karayaka sheep are considered one of the main indigenous sheep breeds naturally adapted to rural local habitat and geography, with an important economic value for regional sheep breeding activities largely performed in Central and Eastern Black Sea Region areas [17,18].

In the study, body weight (BW, kg) was evaluated alongside heart girth (HG, cm), abdominal girth (AG, cm), diagonal body length (DBL, cm), body length (BL, cm), withers height (WH, cm), rump height (RH, cm), hip width (HW, cm), chest width (CW, cm), and body condition score (BCS). All morphometric measurements were collected by the same experienced operator to minimize measurement variability. Each measurement was recorded twice, and the average of the two measurements was used for subsequent statistical analyses. Body weight was measured using a digital animal scale with a weighing capacity of 300 kg and an accuracy of ±0.1 kg. Morphometric measurements were taken in centimeters with the aid of a flexible measuring tape and a measuring stick. Heart girth was measured around the thoracic cage immediately behind the forelimbs; abdominal girth was measured at the widest part of the abdomen; diagonal body length was measured as the distance from the shoulder prominence to the ischial tuberosity. Body length (BL) was measured as the horizontal distance from the point of shoulder to the pin bone (tuber ischii). Withers height was measured as the vertical distance from the ground to the withers point, while rump height was the vertical distance from the ground to the rump point. Hip width was recorded as the distance between the two hip prominences, and chest width as the distance between anatomical reference points in the anterior region of the chest. The body condition score was determined according to a 1–5 scoring system used to assess the animals’ muscle and fat reserves [19].

All measurements were conducted during the same rearing period and using the same measurement protocol. The obtained dataset was utilized for biometric analyses aimed at predicting body weight from morphometric characteristics, as well as for the development of multiple linear regression models and machine learning algorithms.

### 2.2. Statistical Analyses

Prior to commencing the analysis of the dataset, checks for missing observations and outliers were conducted for all variables. Descriptive statistics including mean, standard error, min–max values, and coefficient of variation (%) were applied to body weight (BW) and morphometric traits. Differences between sex groups were determined using an independent samples *t*-test, with a statistical significance level set at *p* < 0.05 [20].

Pearson correlation coefficients were calculated based on the relationships between body weight and morphometric characteristics. Pearson correlation is a simple and commonly used method for estimating linear association between two continuous measures [21]. A correlation matrix and a correlation heatmap were created to visually assess the relationship between variables.

### 2.3. Principal Component Analysis and Multiple Linear Regression

Principal component analysis (PCA) was conducted to examine the shared variation structure among morphometric characteristics and to reveal the fundamental structure of the dataset by reducing the number of variables. PCA is a multivariate statistical method that summarizes a large number of interrelated variables under a smaller number of independent components [22,23]. Prior to the analysis, all variables were standardized, and eigenvalues, explained variance ratios, and component loadings were calculated using the correlation matrix. The Kaiser criterion was considered in the interpretation of principal components, and components with eigenvalues greater than 1 were included in the evaluation [24]. Additionally, PCA biplot graphs were generated to visually examine the relationships between variables and principal components.

Multiple linear regression analysis was applied to predict live weight from morphometric characteristics. Multiple linear regression is one of the fundamental statistical modeling methods that allows the simultaneous evaluation of the relationship between a dependent variable and multiple independent variables [25]. The extent of multicollinearity between the independent variables was assessed using the variance inflation factor (VIF) before building models. Variance inflation factor values are widely used to detect multicollinearity problems and their impact on the stability of regression coefficients [26]. In the last model, regression coefficients, standard errors, t-statistics, and their significance levels were calculated, as well as the coefficient of determination (R^2^), adjusted coefficient of determination, and Durbin–Watson.

### 2.4. Machine Learning Algorithms and the Evaluation of Model Performance

Various machine learning algorithms, in addition to simple regression methods, were tested for the evaluation of body weight predictive performance. Under this scope, Linear Regression, Ridge Regression, LASSO Regression, Random Forest and Gradient Boosting models were experimented on. Prior to model development, predictor variables were standardized using the StandardScaler function to have zero mean and unit variance. This preprocessing step was performed to improve numerical stability and ensure comparable variable scales, particularly for the regression-based models. The dataset was randomly divided into training (80%) and testing (20%) subsets. An independent hold-out validation strategy was adopted to provide a consistent evaluation framework for comparing the predictive performance of all conventional regression and machine learning models using the same previously unseen test dataset. This approach was considered appropriate for ensuring an unbiased comparison of model performance across all evaluated methods. The training dataset was used for model development, whereas model performance was evaluated using the independent testing dataset. The training dataset was used for building the model, while its performance was evaluated on the external test dataset.

The study used a linear regression model as a baseline model for comparison. The Ridge Regression model empirically shrunk coefficient estimates with the addition of an L2 penalty term in order to avoid overfitting and address multicollinearity [27]. An approach like Ridge shrinks coefficients away from zero, but not to exactly zero. In comparison, the LASSO Regression uses an L1 penalty function. The LASSO method is useful for variable selection because it sets the value of some coefficients equal to 0, thus allowing training on more parsimonious and interpretable models, especially with highly correlated explanatory variables [28,29].

The hyperparameters of the machine learning models were determined based on commonly recommended values reported in the literature and preliminary exploratory analyses to achieve stable model performance while avoiding unnecessary model complexity. The same hyperparameter settings were retained throughout all analyses to ensure a fair comparison among the evaluated algorithms.

The Random Forest algorithm, one of the tree-based ensemble learning methods, is based on the aggregation of a large number of decision trees created through the bootstrap sampling technique [11]. Each decision tree is constructed from different random samples of the training dataset, and at each node, randomly selected subsets of explanatory variables are evaluated. The final prediction is calculated by averaging the predictions obtained from all trees. This approach reduces the risk of overfitting and enables the successful modeling of nonlinear relationships. In this study, the Random Forest model was constructed using 500 decision trees.

Gradient Boosting is a boosting-based ensemble learning technique; it refers to reducing prediction errors by the sequential combination of weak learners [12]. This algorithm works on the premise of fitting a decision tree based on the residuals of errors estimated by the previous model and successively enhancing the performance of the model. Among others, Gradient Boosting algorithms are popular and often used in complex biological datasets because they can model nonlinear relationships [30]. Gradient Boosting model was trained on 150 decision trees, with a 0.05 learning rate, a maximum depth of a tree of 2, and a subsample of 0.80 in this study.

Model performance was evaluated using the coefficient of determination (R^2^), root mean square error (RMSE), and mean absolute error (MAE). The coefficient of determination (R^2^) represents the proportion of variation in body weight explained by the model, whereas RMSE quantifies the average magnitude of prediction errors by assigning greater weight to larger errors. MAE represents the average absolute difference between observed and predicted body weight values and provides an easily interpretable measure of prediction accuracy. Higher R^2^ values and lower RMSE and MAE values indicate superior model performance.

In the comparison of model performances, the coefficient of determination (R^2^), root mean square error (RMSE), and mean absolute error (MAE) criteria were utilized. While the R^2^ value indicates the explanatory power of the model, the RMSE and MAE values were employed to assess the magnitude of prediction errors [31]. Performance metrics obtained from the training and test datasets were evaluated collectively to compare the prediction accuracy and generalizability levels of the models.

### 2.5. Explainable Artificial Intelligence Analysis (SHAP)

Variable importance analysis was conducted for the model demonstrating the highest prediction performance. Additionally, SHAP (SHapley Additive Explanations) analysis was applied to enhance the interpretability of the model predictions. SHAP values were calculated using the TreeExplainer algorithm implemented in the SHAP Python library, which is specifically designed for tree-based machine learning models. TreeExplainer computes Shapley values by quantifying the contribution of each predictor variable to the model output relative to the average model prediction. SHAP is an explainable artificial intelligence framework based on Shapley values from cooperative game theory that can break down the contribution of each variable to the model prediction, which is separately calculated [16]. In addition to ranking all variables in order of importance, this also allows us to measure whether the variable is associated with an increase or a decrease in prediction. The SHAP approach has gained considerable attention in recent years as a feature attribution method to provide more interpretability of machine learning models applied in biological data analysis, genomic studies and livestock [15]. In this study, the contribution of each morphometric feature to the prediction of live weight was evaluated in terms of direction and magnitude using SHAP values.

Finally, all data processing, statistical analyses, machine learning applications, and visualization procedures were conducted using Python version 3.12.10. The pandas library was used for data management, NumPy for numerical computations, SciPy and statsmodels for statistical analyses, scikit-learn for data standardization and machine learning applications, Matplotlib and Seaborn for data visualization, and the SHAP library for explainable artificial intelligence analyses. Further information on these software packages is available from the official websites of Python (https://www.python.org/), pandas (https://pandas.pydata.org/), NumPy (https://numpy.org/), SciPy (https://scipy.org/), statsmodels (https://www.statsmodels.org/), scikit-learn (https://scikit-learn.org/), Matplotlib (https://matplotlib.org/), Seaborn (https://seaborn.pydata.org/), and SHAP (https://shap.readthedocs.io/) (all accessed on 11 April 2026).

## 3. Results

### 3.1. Descriptive Statistics and the Effect of the Gender Factor

Table 1 shows the descriptive statistics of body weight and morphometric characteristics of Karayaka lambs, as well as independent-samples t-test results for the gender factor. Statistically significant differences (*p* < 0.05) were identified between sexes for all traits studied. The average body weights of male and female lambs were determined as 30.49 ± 0.38 kg and 28.49 ± 0.24 kg, respectively (*p* < 0.001). Male lambs exhibited significantly higher values for HG, AG, DBL, BL, WH, RH, HW, CW, and BCS than female lambs. The coefficients of variation in the variables ranged from 3.35 to 10.03%, with the highest variability for body weight and body length showing low variation.

### 3.2. Results of Correlation Analysis and Principal Component Analysis

**Figure 1:**
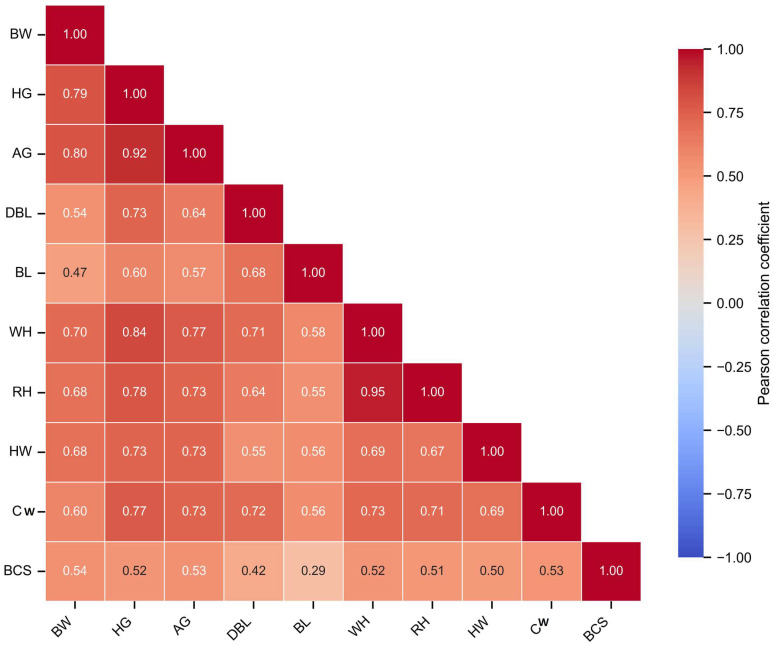
Pearson correlation matrix among body weight and morphometric traits in Karayaka lambs.

In order to determine the relationships between body weight and morphometric characteristics, Pearson correlation analysis was conducted, and the results are presented in Table 2 and Figure 1. Positive correlations were identified between body weight and all examined morphometric traits. The highest correlations with body weight were found between abdominal girth (r = 0.796) and heart girth (r = 0.794). Additionally, very strong positive correlations were detected between withers height and rump height (r = 0.947), as well as between heart girth and abdominal girth (r = 0.921). In contrast, the relationship between body weight and body length was relatively lower (r = 0.470).

**Figure 2:**
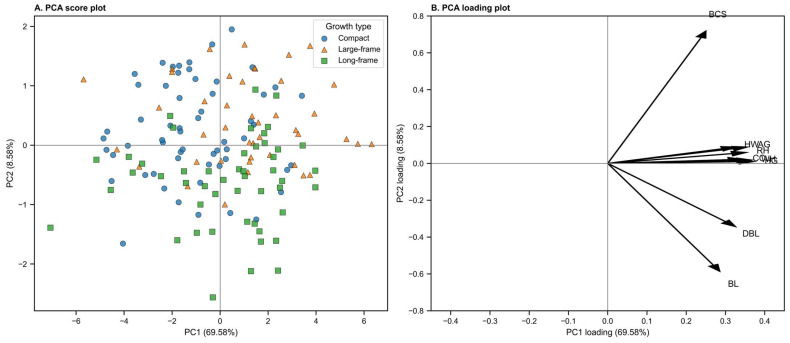
Principal component analysis (PCA) biplot of morphometric traits in Karayaka lambs.

**Figure 3:**
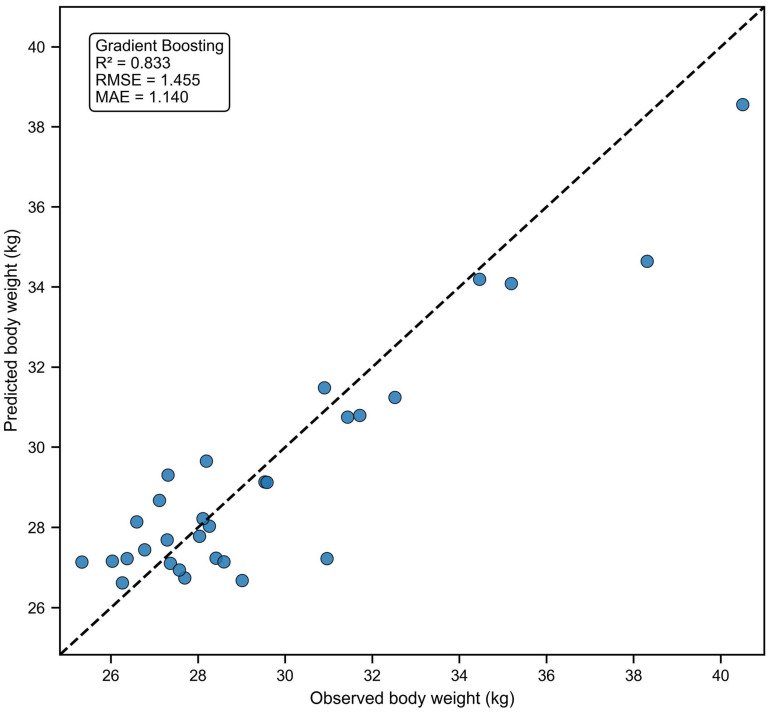
Relationship between observed and predicted body weight values in Karayaka lambs based on the Gradient Boosting model.

Principal component analysis (PCA) was performed to evaluate the multivariate structure of morphometric characteristics, and the results are shown in Table 3 and Figure 2. The first principal component (PC1) accounted for 69.58% of the total variance with an eigenvalue of 6.305. The second and the third components explained 8.58% and 5.77% of the variance, respectively. The composite of the first three principal components accounted for 83.94% of the total variance. The component loadings showed that PC1 was mainly constituted by heart girth (0.372), withers height (0.366), abdominal girth (0.356), rump height (0.353) and chest width (0.344). These results indicate that the first principal component primarily represents overall body size. The PCA biplot also showed that the directions of circumference and height measurements were similar, again suggesting this morphological variation is largely due to scaling of the body. Figure 3 presents the relationship between the observed and predicted body weight values obtained using the Gradient Boosting model.

### 3.3. Multiple Linear Regression Analysis

In order to identify the most significant variables that can be used in the prediction of body weight, variance inflation factor (VIF) analysis and multiple linear regression analysis were conducted. The final regression model and VIF results are presented in Table 4. Following the assessment of multicollinearity, abdominal girth (AG), withers height (WH), hip width (HW), and body condition score (BCS) were included in the model. The resulting regression equation was determined as shown in Equation (1):

BW = −25.405 + 0.400 (AG) + 0.194 (WH) + 0.597 (HW) + 1.277 (BCS)(1)

The model explained 67.7% of the total variation in body weight (R^2^ = 0.677; Adjusted R^2^ = 0.668). The highest contribution to the model was provided by abdominal girth (β = 0.400; *p* < 0.001), followed by hip width (β = 0.597; *p* = 0.044) and body condition score (β = 1.277; *p* = 0.042). Withers height demonstrated borderline significance (*p* = 0.068). Variance inflation factor (VIF) values ranged from 1.559 to 14.228, with the highest value observed for withers height.

### 3.4. Comparison of Machine Learning Models

In order to compare the body weight prediction performances of traditional statistical methods and machine learning algorithms, linear regression, Ridge Regression, LASSO Regression, Random Forest, and Gradient Boosting models were implemented. The results obtained are presented in Table 5. Among the models, the highest prediction accuracy was achieved by the Gradient Boosting algorithm. This model reached R^2^ = 0.962 on the training data and R^2^ = 0.862 on the test data, while also exhibiting the lowest error values (RMSE = 1.320 kg; MAE = 1.034 kg). The Random Forest model ranked second with an R^2^ value of 0.828 on the test data. In contrast, the performances of the linear regression, Ridge, and LASSO models on the test dataset were found to be quite similar, with R^2^ values calculated at approximately 0.736. The results indicate that ensemble-based machine learning algorithms provide higher prediction accuracy compared to traditional regression methods.

### 3.5. Variable Importance and SHAP Analyses

**Figure 4:**
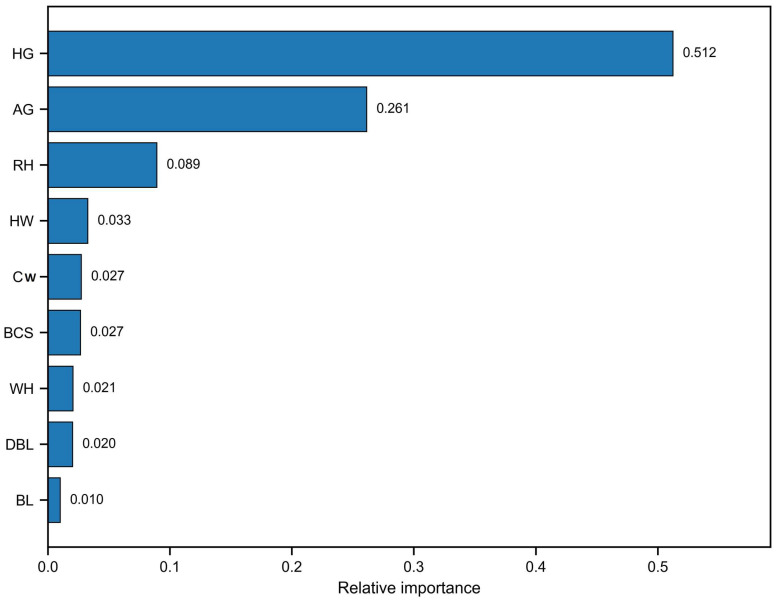
Relative importance of morphometric traits for body weight prediction based on the Gradient Boosting model.

**Figure 5:**
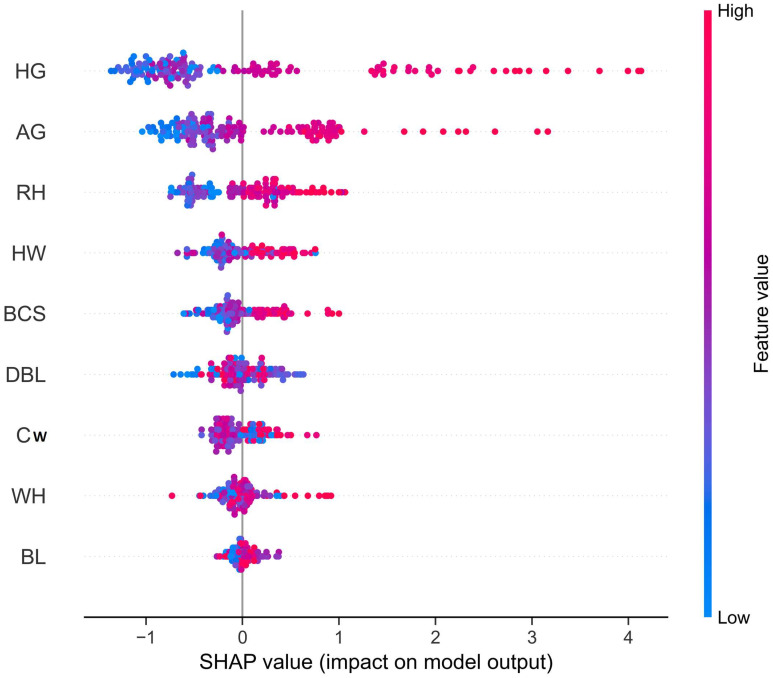
SHAP summary plot illustrating the contribution of morphometric traits to body weight prediction in Karayaka lambs.

In order to enhance the interpretability of the highest-performing model, variable importance analysis and SHAP (SHapley Additive exPlanations) analysis were conducted. The results are presented in Figure 4 and Figure 5, respectively. According to the variable importance ranking, heart girth was identified as the most influential variable in predicting body weight, accounting for 51.24% of the total importance. This was followed by abdominal girth with an importance level of 26.12%. Rump height (8.92%), hip width (3.26%), chest width (2.73%), body condition score (2.67%), withers height (2.05%), diagonal body length (2.02%), and body length (1.00%) had lower contributions. The SHAP analysis results corroborated the dominant influence of heart and abdominal girth on the model predictions; higher values of these features were found to increase the body weight estimates, whereas lower values decreased them.

## 4. Discussion

The present study demonstrated that male Karayaka lambs exhibited significantly higher body weight and morphometric measurements than female lambs. Sex is one of the major biological factors influencing growth and body development in sheep, and differences between males and females become increasingly evident as animals approach maturity. Previous studies have consistently reported that male lambs generally achieve greater body weight and larger body dimensions than female lambs due to differences in growth rate, hormonal regulation, and muscle development [32,33]. The findings obtained in the present study are therefore consistent with previous reports indicating superior growth performance in male sheep. Furthermore, the relatively low coefficients of variation observed across traits suggest that the experimental population was morphologically homogeneous, thereby increasing the reliability of the subsequent biometric and machine learning analyses.

Correlation analysis revealed significant positive relationships between body weight and all morphometric characteristics. Among the evaluated traits, abdominal girth and heart girth exhibited the strongest associations with body weight. These findings are in agreement with numerous previous studies reporting that circumference-related measurements represent the most reliable indicators of body weight in sheep populations [32,34,35]. Similar observations have also been reported for Karayaka sheep, where chest and body circumference measurements were found to be strongly associated with body weight and growth performance [18]. From a biological perspective, circumference measurements provide a more comprehensive representation of overall body volume, skeletal development, and muscle deposition than linear measurements alone. Consequently, they often outperform body length or height measurements when predicting body weight.

Principal component analysis further demonstrated that most morphometric variables were strongly associated with a common body size component. The first principal component explained approximately 70% of the total variation and was characterized by high positive loadings for heart girth, abdominal girth, withers height, rump height, and chest width. Similar findings have been reported in previous studies investigating the morphological structure of sheep populations using PCA [9,10,36]. In addition, ref. [37] reported that the first principal component explained a substantial proportion of total variation in Zulu sheep and primarily represented general body size and conformation. The clustering of circumference and height measurements within the same principal component observed in the present study indicates that these traits are biologically interconnected and collectively reflect overall growth and structural development in Karayaka lambs.

The multiple linear regression model identified abdominal girth, withers height, hip width, and body condition score as significant predictors of body weight. The model explained approximately 68% of the observed variation, indicating that morphometric traits provide substantial information for body weight estimation. Although withers height showed a relatively high VIF value, it was retained in the final model because height-related measurements are biologically linked to skeletal development and overall body size, and its inclusion improved the explanatory capacity of the model. Previous studies have similarly reported that circumference measurements, body length, and height-related traits are among the most important predictors of body weight in sheep [2,32,38].

Among the evaluated predictive approaches, the Gradient Boosting algorithm produced the highest prediction accuracy, followed by Random Forest. Both ensemble-based machine learning algorithms substantially outperformed conventional regression methods. The superior performance of these models can be attributed to their ability to capture nonlinear relationships and complex interactions among explanatory variables. Previous studies have similarly reported that Random Forest, Gradient Boosting, and related ensemble learning techniques achieve higher predictive performance than traditional regression models when estimating body weight in sheep and other livestock species [13,14,39,40]. Although linear regression, Ridge Regression, and LASSO Regression provided acceptable predictive performance, their lower test R^2^ values indicate that the relationship between body weight and morphometric traits cannot be fully explained using simple linear assumptions. Consequently, machine learning algorithms appear better suited for modeling the complex biological processes underlying body growth.

The predictive performance obtained in the present study is generally consistent with recent machine learning studies conducted on different sheep breeds. Ref. [14] reported that Random Forest achieved a test R^2^ of 0.873 (RMSE = 1.479 kg; MAE = 1.105 kg) for predicting body weight in Blackbelly sheep using biometric measurements. Similarly, ref. [40] demonstrated the successful application of machine learning algorithms for predicting weaning weight in Romanov lambs, while [13] highlighted the effectiveness of ensemble learning methods for body weight estimation in Peruvian Corriedale sheep. Although direct comparisons among studies should be interpreted cautiously because of differences in breed, age, sample size, management conditions, and predictor variables, these findings collectively indicate that ensemble-based machine learning algorithms consistently outperform conventional regression models in body weight prediction.

Variable importance and SHAP analyses provided additional insight into the biological interpretation of model predictions. Heart girth and abdominal girth emerged as the two most influential predictors, accounting for the majority of the model’s explanatory power. Furthermore, SHAP values demonstrated that increases in these measurements consistently contributed to higher predicted body weight values. These findings were highly consistent with the results obtained from correlation analysis and regression modeling, thereby reinforcing the importance of circumference-related traits in body weight estimation. The application of SHAP analysis is particularly valuable because it enables interpretation of complex machine learning models while maintaining predictive accuracy [15,16]. As a result, explainable artificial intelligence approaches can enhance confidence in model predictions and facilitate biological interpretation of machine learning outcomes.

The principal strength of the proposed framework lies in the integrated evaluation of conventional regression methods, machine learning algorithms, and SHAP-based explainable artificial intelligence within a single analytical workflow. This approach not only improves predictive performance but also provides biological interpretability by identifying the relative contribution of individual morphometric traits. Nevertheless, several limitations should be acknowledged. The present study was based on animals raised in a single commercial flock and within a relatively narrow age range. In addition, external validation using independent populations was not performed, and environmental and nutritional factors were not incorporated into the prediction models. Therefore, further validation using independent populations from different breeds and production systems would be valuable for confirming the robustness and generalizability of the proposed framework.

Although the proposed prediction framework demonstrated promising performance in Karayaka lambs, its applicability to other sheep breeds, management systems, and production environments should be interpreted with caution. The present study was conducted using a relatively homogeneous population comprising lambs from a single commercial flock within a narrow age (160–200 days) and body weight range. Consequently, the developed models may not fully represent the biological variability present in larger and more diverse sheep populations. Furthermore, environmental, nutritional, and management-related factors were not incorporated into the prediction models. Therefore, external validation using independent populations representing different breeds, age groups, body weight ranges, and production systems is necessary before the proposed models can be generalized to broader sheep populations.

Overall, the findings of the present study demonstrate that morphometric measurements can be effectively utilized for body weight estimation in Karayaka lambs. These measurements are easy to obtain under field conditions and therefore provide practical alternatives when weighing equipment is unavailable.

## 5. Conclusions

Morphometric measurements can be effectively used for accurate body weight prediction in Karayaka lambs using both conventional statistical methods and machine learning algorithms. Among the evaluated models, the Gradient Boosting algorithm demonstrated the highest predictive performance, indicating its potential as a reliable tool for practical body weight estimation. Furthermore, the integration of machine learning with explainable artificial intelligence improved model interpretability by identifying the most influential morphometric predictors. Although the proposed framework showed promising performance in Karayaka lambs, further validation using independent datasets representing different flocks, age groups, and production environments is warranted to confirm the robustness and generalizability of the developed models.

## Figures and Tables

**Table 1:** Descriptive statistics of body weight and morphometric traits according to sex in Karayaka lambs.

Variable	Female (Mean ± SE)	Male (Mean ± SE)	Min–Max	CV (%)	*p*-Value
BW	28.49 ± 0.24	30.49 ± 0.38	24.59–40.50	10.03	<0.001
HG	73.90 ± 0.36	75.09 ± 0.37	65.69–83.45	4.33	0.022
AG	75.12 ± 0.40	76.74 ± 0.46	65.15–88.81	5.01	0.008
DBL	48.29 ± 0.22	49.01 ± 0.24	42.62–53.02	4.09	0.025
BL	44.56 ± 0.17	45.20 ± 0.17	41.20–48.63	3.35	0.008
WH	61.23 ± 0.25	62.01 ± 0.25	55.84–67.96	3.57	0.03
RH	62.16 ± 0.25	63.10 ± 0.27	56.75–69.11	3.66	0.012
HW	13.79 ± 0.08	14.21 ± 0.08	12.19–15.96	5.27	<0.001
CW	7.28 ± 0.04	7.42 ± 0.04	6.15–8.07	4.48	0.007
BCS	3.20 ± 0.03	3.40 ± 0.03	2.58–4.01	8.28	<0.001

BW: Body weight (kg); HG: Heart girth (cm); AG: Abdominal girth (cm); DBL: Diagonal body length (cm); BL: Body length (cm); WH: Withers height (cm); RH: Rump height (cm); HW: Hip width (cm); CW: Chest width (cm); BCS: Body condition score. SE: Standard error of the mean; CV: Coefficient of variation. *p*-values were obtained from independent samples t-tests comparing female and male lambs.

**Table 2:** Pearson correlation coefficients among body weight and morphometric traits in Karayaka lambs.

Variable	BW	HG	AG	DBL	BL	WH	RH	HW	CW	BCS
BW	1									
HG	0.794	1								
AG	0.796	0.921	1							
DBL	0.544	0.731	0.635	1						
BL	0.47	0.602	0.566	0.684	1					
WH	0.703	0.843	0.767	0.714	0.583	1				
RH	0.68	0.784	0.728	0.645	0.554	0.947	1			
HW	0.682	0.73	0.728	0.553	0.559	0.694	0.667	1		
CW	0.601	0.767	0.729	0.718	0.558	0.725	0.711	0.687	1	
BCS	0.541	0.523	0.532	0.417	0.288	0.518	0.512	0.499	0.529	1

BW: Body weight; HG: Heart girth; AG: Abdominal girth; DBL: Diagonal body length; BL: Body length; WH: Withers height; RH: Rump height; HW: Hip width; CW: Chest width; BCS: Body condition score. All correlation coefficients were significant at *p* < 0.05.

**Table 3:** Eigenvalues, explained variance, cumulative variance, and loading coefficients of the principal component analysis (PCA) performed on morphometric traits of Karayaka lambs.

Trait/Component	PC1	PC2	PC3
Eigenvalue	6.305	0.778	0.523
Variance explained (%)	69.583	8.582	5.774
Cumulative variance (%)	69.583	78.165	83.939
HG	0.372	0.01	−0.197
AG	0.356	0.089	−0.197
DBL	0.326	−0.344	0.333
BL	0.286	−0.587	0.442
WH	0.366	0.018	−0.331
RH	0.353	0.059	−0.373
HW	0.327	0.089	−0.074
CW	0.344	0.022	0.109
BCS	0.25	0.719	0.592

HG: Heart girth; AG: Abdominal girth; DBL: Diagonal body length; BL: Body length; WH: Withers height; RH: Rump height; HW: Hip width; CW: Chest width; BCS: Body condition score.

**Table 4:** Multiple regression model and multicollinearity diagnostics for body weight prediction in Karayaka lambs.

Variable	Regression Coefficient (β)	SE	*t*-Value	*p*-Value	VIF
Intercept	−25.405	3.945	−6.44	<0.001	—
AG	0.4	0.064	6.241	<0.001	7.377
WH	0.194	0.105	1.842	0.068	14.228
HW	0.597	0.293	2.037	0.044	2.664
BCS	1.277	0.623	2.049	0.042	1.559
Statistic		Value			
R^2^		0.677			
Adjusted R^2^		0.668			
F-statistic		75.86			
*p*-value (model)		<0.001			

AG: Abdominal girth; WH: Withers height; HW: Hip width; BCS: Body condition score; VIF: Variance inflation factor.

**Table 5:** Comparison of conventional statistical and machine learning models for body weight prediction in Karayaka lambs.

Model	Train R^2^	Test R^2^	RMSE (kg)	MAE (kg)
Gradient Boosting	0.962	0.862	1.32	1.034
Random Forest	0.97	0.828	1.476	1.087
LASSO Regression	0.668	0.736	1.827	1.364
Linear Regression	0.669	0.736	1.828	1.366
Ridge Regression	0.668	0.736	1.829	1.358

Note: R^2^: coefficient of determination; RMSE: root mean square error; MAE: mean absolute error.

## Data Availability

The data presented in this study are available from the corresponding author upon reasonable request.
